# In Search of a Cure: The Development of Therapeutics to Alter the Progression of Spinal Muscular Atrophy

**DOI:** 10.3390/brainsci11020194

**Published:** 2021-02-05

**Authors:** Kristine S. Ojala, Emily J. Reedich, Christine J. DiDonato, Stephen D. Meriney

**Affiliations:** 1 Department of Neuroscience, University of Pittsburgh, Pittsburgh, PA 15260, USA; k.ojala@pitt.edu; 2 Human Molecular Genetics and Physiology Program, Stanley Manne Children’s Research Institute at Ann & Robert H. Lurie Children’s Hospital, Chicago, IL 60611, USA; ereedich@uri.edu (E.J.R.); c-didonato@northwestern.edu (C.J.D.); 3 Department of Pediatrics, Feinberg School of Medicine, Northwestern University, Chicago, IL 60611, USA

**Keywords:** spinal muscular atrophy, motoneuron disease, neuromuscular disease, therapeutics

## Abstract

Until the recent development of disease-modifying therapeutics, spinal muscular atrophy (SMA) was considered a devastating neuromuscular disease with a poor prognosis for most affected individuals. Symptoms generally present during early childhood and manifest as muscle weakness and progressive paralysis, severely compromising the affected individual’s quality of life, independence, and lifespan. SMA is most commonly caused by the inheritance of homozygously deleted *SMN1* alleles with retention of one or more copies of a paralog gene, *SMN2*, which inversely correlates with disease severity. The recent advent and use of genetically targeted therapies have transformed SMA into a prototype for monogenic disease treatment in the era of genetic medicine. Many SMA-affected individuals receiving these therapies achieve traditionally unobtainable motor milestones and survival rates as medicines drastically alter the natural progression of this disease. This review discusses historical SMA progression and underlying disease mechanisms, highlights advances made in therapeutic research, clinical trials, and FDA-approved medicines, and discusses possible second-generation and complementary medicines as well as optimal temporal intervention windows in order to optimize motor function and improve quality of life for all SMA-affected individuals.

## 1. Genetics

Over the last 129 years, physicians and researchers have made substantial progress in recognizing, understanding, and treating the autosomal recessive genetic disorder spinal muscular atrophy (SMA). Although universally distinguishable by the pathological loss of lower α-motoneurons (most often during infancy or adolescence), SMA-affected individuals can present with a clinically heterogenous spectrum of symptoms. The variable presentation of symptoms undoubtedly obfuscated the recognition of the singular and unifying etiology of this disorder for the first 100 years after initial documentation. Generally, motor symptoms appear as symmetrical, predominantly proximal and axial muscle weakness and range from fatal somatic and respiratory paralysis to minor gait abnormalities. In contrast to the diverse presentation of this disorder, SMA is simply a complex manifestation of a relatively straightforward problem.

**Figure 1:**
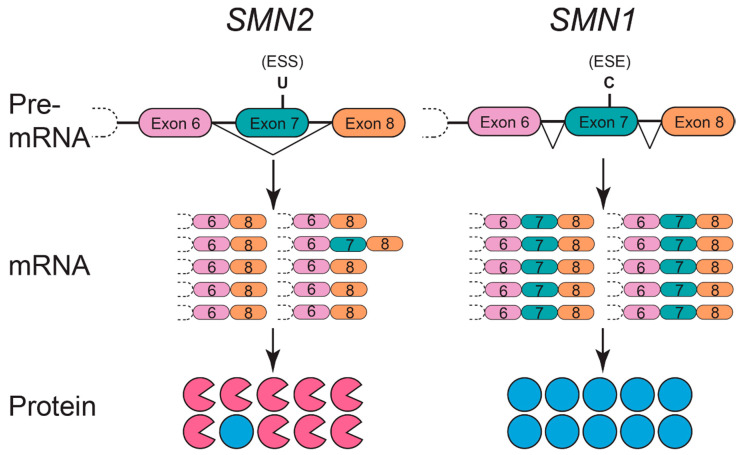
Differential pre-mRNA splicing of *SMN1* and *SMN2* genes. The *SMN1* gene (right) effectively splices exon 7 into the mature mRNA transcript, resulting in ~100% full-length SMN protein (blue full circles). In contrast, a C to T nucleotide transition in exon 7 of the *SMN2* gene (left; resulting in a U nucleotide in exon 7 pre-mRNA) causes ~90% of the mature mRNA transcripts to lack exon 7 (pink incomplete circles). Without exon 7, the truncated protein (SMNΔ7) is unstable, ineffective at oligomerization, and consequently degraded.

SMA is a monogenic, autosomal recessive disorder caused by the homozygous functional loss or deletion of a 5q13 gene critical for viability of motoneurons (aptly named *survival motor neuron*, or specifically in higher primates, *SMN1*) [1]. The absence of SMN protein results in embryonic lethality [2]; thus, SMN is an essential protein for development. A single copy of the *SMN* gene is phylogenetically conserved across most eukaryotes, but humans are uniquely fortunate to possess a second *SMN* gene. The range in clinical symptoms is generally attributed to variable expression of this genetic paralog, *survival motor neuron 2* (*SMN2*). The paramount difference in the nucleotide sequence of *SMN1* versus *SMN2* is a translationally silent cytosine (C) to thymine (T) nucleotide transition at the sixth position in *SMN2* exon 7 [3,4]. This nucleotide transition modifies *SMN2* pre-mRNA splicing and promotes exon 7 exclusion from the mature transcript (Figure 1). *SMN2* exon 7 skipping occurs because the C to T transition impedes an exonic splicing enhancer (ESE) site to which SF2/ASF binds [5] and creates a novel exonic splicing silencer (ESS) site to which the splicing factors hnRNP A1/A2 bind [6]. The alternatively spliced SMNΔ7 protein is unstable, inefficiently self-oligomerizes and is subsequently degraded [7]. While *SMN1* selectively generates full-length SMN protein, only ~10 percent of protein produced by *SMN2* is full-length SMN, and the remaining 90 percent is the less-functional SMNΔ7 splice variant that is rapidly eliminated. Due to the low production of functional SMN protein, *SMN2* can only partially compensate for loss of *SMN1*, and thus SMA arises from a deficiency but not complete depletion of SMN protein.

Curiously, *SMN2* copy number varies in the human population and ranges from 0 to 8 copies. This variability is likely due to the characteristic instability of the chromosome 5q13 region [8]. Each extra *SMN2* copy at the *SMN* locus serves to bolster the amount of functional, full-length SMN protein generated within a cell. This natural variability in *SMN2* expression in the absence of *SMN1* permits the clinical SMA severity to exist along a spectrum predominantly determined by *SMN2* copy number [9,10,11,12].

## 2. Clinical Manifestations

Clinical features that define SMA include progressive muscle weakness, hypotonia, and atrophy of skeletal muscle resulting from degeneration and loss of spinal motoneurons [13,14]. Given the wide range of clinical phenotypes, SMA has been traditionally classified into five categories (Type 0 to Type IV) based on age at symptom onset and highest motor milestone achieved (Table 1) [15,16,17,18]. Generally, the clinical subtype inversely correlates with *SMN2* copy number and may provide physicians with some clinical insight and prognostic information.

The natural history of each clinical subtype (reviewed in [19]) is described as follows: in all subtypes, proximal muscle groups are more affected than distal muscle groups, with legs more affected than arms. The most severe form of SMA (Type 0) may present with prenatal onset (reduced movement in utero) and newborns may be born unable to swallow and breathe independently. These infants exhibit severe muscle weakness and lack of tone, joint contractures, areflexia, bilateral facial paralysis and early respiratory failure [20,21]. Outside of bulbar and spinal motoneuron dysfunction, thalamus, heart, vasculature, and sensory system may also develop problems due to exceedingly low systemic SMN levels [22,23,24,25,26]. Type 0 SMA patients carry a single copy of *SMN2* and have life expectancies of less than six months. 

Approximately half of all SMA cases are classified as Type I (Werdnig–Hoffman disease). Infants with Type I SMA exhibit symptom onset prior to six months of age and are unable sit unassisted due to proximal muscle weakness and hypotonia [27]. This weakness manifests as a frog-leg posture, poor head control, paradoxical breathing, bulbar involvement (tongue fasciculation and dysphagia), reduced or absent reflexes, and respiratory failure before the age of two. Without respiratory, nutritional, or genetically targeted interventions, life expectancy is less than two years of age [28]. Type I SMA patients typically have two copies of *SMN2*. Although Type I SMA has the highest incidence of the five clinical subtypes, milder forms of the disorder are historically more prevalent in the population due to the increased lifespan of intermediate and mild SMA patients (Type II–IV). However, the advent and use of genetically targeted therapies for SMA will dramatically shift this distribution.

Patients with Type II SMA (Dubowitz disease) manifest motor symptoms between six and 18 months of age. These patients may sit without assistance but do not independently stand or achieve ambulation [29]. Type II SMA patients display progressive proximal muscle weakness, lack muscle tone, and present with diminished reflexes. Respiratory dysfunction is common, and the development of scoliosis plus weakened intercostal muscles can affect pulmonary function. Type II SMA patients develop hand tremors (polyminimyoclonus), contractures, and occasionally ankylosis of the mandible [13]. These patients typically have three copies of *SMN2* and reduced life expectancy.

Patients with Type III SMA (Kugelberg–Welander disease) experience initial symptom onset during early childhood but substantial loss of ambulation generally occurs during puberty [30]. These individuals present with progressive proximal muscle weakness that is greater in the legs than the arms, and may retain the ability to walk but with considerable difficulty due to gait abnormalities and muscle atrophy/weakness [19]. However, some of these patients irreversibly lose walking ability. Type III SMA patients may develop polyminimyoclonus, but respiratory dysfunction and severe scoliosis are not components of the clinical description. Patients typically have 3–4 copies of *SMN2* and a normal or near-normal life expectancy.

Lastly, patients with Type IV SMA (<5% of cases) develop symptoms in adulthood and experience the mildest disease course (generally restricted to gait abnormalities), which is attributed to them usually having ≥ 4 *SMN2* copies [18,31]. While clinical subcategorization of SMA is useful in guiding treatment strategies and management of medical and developmental expectations, it is crucial to recognize that the clinical spectrum of SMA severity exists on a continuum and that division into subtypes may not fully capture the experience and expectations of patients, their families, and their health care providers. In addition, the recent advancements in disease-modifying SMA therapies have substantially altered the expectations of categorical patients, resulting in patients transcending subtypes. As SMN-dependent and -independent therapies continue to improve and become widely accessible to SMA patients, traditional classifications will need to be improved to capture previously unidentifiable symptoms and outcome expectations revealed by prolonged lifespans and improved motor function.

## 3. Impact of Motor Impairment on Quality of Life

SMA-affected individuals experience a slow and progressive decline in motor function and report limitations arising from difficulties in mobility, daily activity, and pervasive fatigue associated with deteriorating physical health [32,33]. Though the initial severity of SMA symptoms (and thus the maximal motor functions achieved) is variable, depreciation of function results in considerable physical disability for all SMA-affected individuals. Inadequate motor function may require daily tasks to be performed by a caregiver in order to help maintain or enhance an individual’s autonomy. These tasks include activities such as meal preparation and hygiene maintenance and are usually informally provided by relatives. One Spanish study evaluating the economic burden incurred by SMA-affected individuals and their caregivers found that the invisible “cost” (compared to medical cost) of informal caregiving accounts for more than two-thirds of the total annual cost of healthcare associated with SMA [34]. This cost is one of the reasons why parents of individuals with SMA declare that a critical gap in patient needs is the lack of support for activities associated with daily living [35].

In addition to motor impairment, patients also report emotional difficulties, which thus far lack effective interventions. These difficulties are due in part to the immense psychosocial burdens experienced by those living with SMA. In addition to the need to make difficult treatment choices, stress, limitations on social activities, and a lack of independence, the pervasive fear of losing functional ability significantly contributes to substantial negative mental health experienced by patients and their families [36]. When surveyed on which tasks would meaningfully improve quality of life, SMA patients reported that improved ability to do daily tasks such as eating, bathing, grooming, using the restroom, independent transfers to and from wheelchairs, being able to spend time independently, and typing on keyboards would be immensely beneficial [37]. The ability to independently perform these often-underappreciated motor tasks should be a goal for the treatment of SMA, and small changes may be sufficient to significantly improve quality of life. Patient, caregiver, and clinician perspectives indicate that even minor improvements in motor function would constitute a meaningful change in disorder outcome [38].

Until the recent development of genetic therapies, management of SMA was traditionally limited to long-term and multi-disciplinary medical, nutritional, and supportive care [39,40,41,42] to alter the natural disorder progression. The advancement of genetic therapies is reshaping the therapeutic environment by mitigating the need for invasive ventilation and extensive medical care, ameliorating progressive motor degeneration, and extending lifespan. However, key outcomes of clinical trials and therapeutic use indicate that impairments in motor function persist in some patient populations [43,44,45,46,47,48,49]. Additionally, not all patient populations are currently able to access or receive genetic therapies. Advances in supportive and therapeutic care have changed the diagnosis of SMA from devastating to hopeful, but complementary treatment approaches are required to further improve the quality of life experienced by SMA-affected individuals and their caregivers.

## 4. The SMN Protein

The SMN protein is ubiquitously expressed in eukaryotic cells, with the highest levels of expression in brain, liver, kidney, and spinal cord cells and moderate levels of expression in cardiac and skeletal muscle cells [50,51]. The full-length human SMN protein is comprised of 294 amino acids, has a molecular weight of 38 kDa, and contains four protein domains (reviewed in [52]): (1) an N-terminal lysine-rich domain (encoded by exons 2A and 2B) responsible for GEMIN2 and nucleic acid binding; (2) a central Tudor domain (encoded by exon 3) that mediates numerous interactions with arginine-glycine (RG)-rich proteins such as one family of core small nuclear ribonucleoproteins (snRNPs) called “Sm” proteins [53] (named by their reactivity with autoantibodies of the Sm serotype from patients with systemic lupus erythematosus [54]); (3) a C-terminal proline-rich domain (encoded by exons 4–6) that is responsible for binding profilin proteins; and (4) a tyrosine-glycine (YG) box (encoded by exon 6) that, along with the last sixteen residues encoded by exon 7, mediates SMN self-oligomerization [7]. Most of these protein domains are conserved amongst vertebrates. Interestingly, it was recently shown that removal of SMN exon 2B can result in a functional SMN protein that restores snRNP function and rescues cell lethality [55]. This implies that GEMIN2 binding is further refined to SMN exon 2A [55].

**Figure 2:**
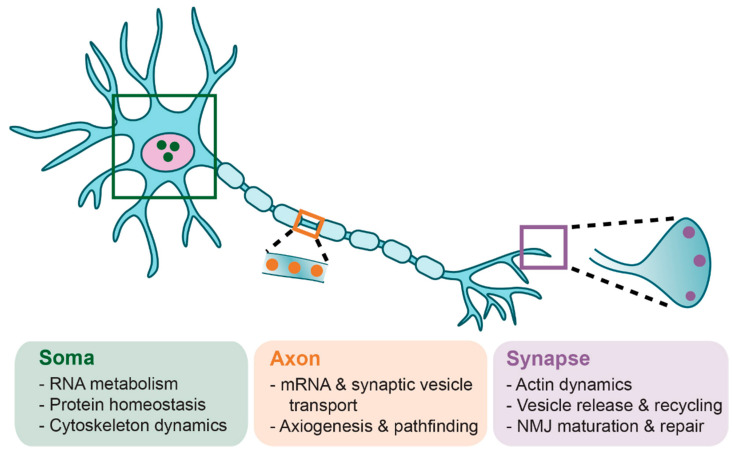
General cellular functions of SMN in motoneurons. SMN has several proposed functions in the motoneuron, though which roles are associated with which specific pathologies are unclear. SMN functions in RNA metabolism, protein homeostasis, and cytoskeleton dynamics. SMN plays additional roles in axonal transport of mRNA and synaptic vesicles, axiogenesis, and axon pathfinding. At the neuromuscular synapse, SMN is involved in actin dynamics, vesicle release and recycling, and neuromuscular junction (NMJ) maturation and repair. These cellular functions of SMN have been thoroughly discussed elsewhere [52,56,70,71,72,73,74].

SMN exhibits both cytoplasmic and nuclear subcellular localization where the protein serves several proposed functions (Figure 2). SMN is a crucial component of snRNP biogenesis [56,57,58]. snRNPs are RNA–protein complexes that are critical constituents of spliceosomes, which recognize and remove introns from pre-mRNA. Cytoplasmic SMN acts in conjunction with GEMIN2-8 and UNR-interacting protein (UNRIP) as the SMN complex to load Sm proteins into a heptameric ring structure on small nuclear RNA (snRNA) [57,59,60,61,62,63,64,65,66,67,68,69]. After further snRNP processing, the SMN complex (still associated with the snRNP molecule) binds snurportin and importin and is transported into the nucleus. The SMN complex and associated snRNP molecules localize to Cajal bodies where snRNP maturation is completed. Other studies suggest that SMN may have numerous additional functions in RNA metabolism, protein homeostasis, and cytoskeletal dynamics (reviewed in [52,70,71]).

While the canonical and most defined role of SMN is snRNP assembly in the soma, several studies have proposed additional, non-canonical roles outside of the soma. In axons, SMN modulates axiogenesis and axonal pathfinding [72], and is involved in axonal transport of mRNA and synaptic vesicles [75,76,77,78]. At the synapse, SMN is involved in local translation of cytoskeletal proteins within presynaptic compartments [78,79]. At the growth cone and synaptic terminal of motoneurons, SMN regulates endocytosis and cytoskeleton activity through its interaction with profilins, a family of proteins regulating actin dynamics [78,80,81,82] and through this interaction has been proposed to regulate the change in ratio of G-actin to F-actin [83], a critical feature for neurite outgrowth [84]. After neuromuscular synapses have matured, SMN contributes to compensatory axonal sprouting following motor nerve injury [85]. It is prudent to note that it remains unclear how these proposed, non-canonical roles of SMN potentially contribute to SMA pathogenesis and symptomology, and more investigation is necessary to definitively understand the extent to which, if any, these potential non-splicing functions of SMN influence the SMA phenotype.

Although additional function(s) of SMN beyond its role in spliceosomal assembly are still debated, the fact that spinal motoneurons are preferentially susceptible to degeneration and death in response to SMN deficiency is quite clear. This has been firmly established through a series of genetic studies in which Smn levels are either specifically reduced or increased within motoneurons [86,87,88,89,90]. However, molecular mechanisms explaining the preferential vulnerability of spinal motoneurons to SMN deficiency are unknown. Three major hypotheses have been posited [71,72,91]. First, SMN deficiency may disrupt splicing of transcripts specifically essential for the function and survival of spinal motoneurons. This notion is supported by studies demonstrating that SMN deficiency differentially alters the snRNA composition of snRNPs across tissue types, which leads to tissue-specific patterns of alternative splicing [92,93]. Second, of all cell types, spinal motoneurons may be most sensitive to systemic splicing defects induced by SMN deficiency. It is possible that the requirement for SMN is uniquely high in spinal motoneurons, and thus even small reductions in SMN result in a deficiency leading to preferential vulnerability to degeneration. Third, SMN may have special spinal motoneuron-specific function(s) that render these cells particularly susceptible to neurodegeneration as a consequence of SMN deficiency.

## 5. Temporal Requirements of SMN

**Figure 3:**
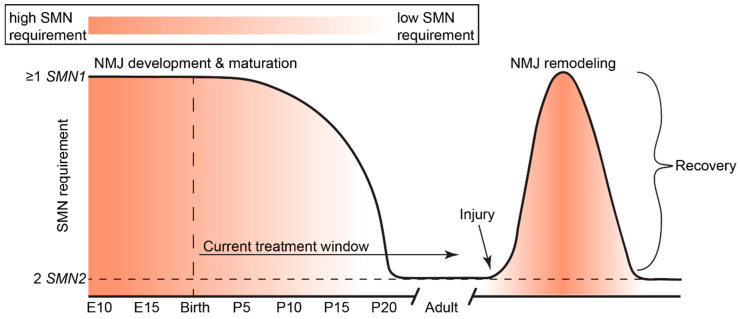
Developmental regulation and requirement of SMN expression in mice. SMN expression is highest during the perinatal period (indicated by an orange to white gradient), but precipitously drops after development of neuromuscular junctions (NMJs; at approximately 17–20 days of age). After the critical period of neuromuscular development, NMJs are viable with low SMN levels. In adulthood, remodeling after injury requires elevated SMN levels to recover and stabilize NMJs. Current therapies for SMA are restricted to the postnatal period. Schematic adapted with permission from Kariya et al., 2014 [85].

In humans and mammals, SMN expression levels are highest during embryonic and early postnatal development, sharply followed by a decrease to a basal level that is maintained throughout life (Figure 3) [94,95]. However, the complex mechanisms regulating this dynamic expression are not well understood. Peak SMN protein levels in the spinal cord are highest during the developmental window of axon sprouting from the spinal cord during early embryogenesis [96,97] and coincide with the onset of myelination [94]. In mice, maximal SMN expression occurs during embryonic day (E)10–13 to permit the growth and pathfinding of motoneuron growth cones to contact target muscles [97]. SMN undergoes an initial decline in expression between E14 and E19, after the developmental period of motoneuron innervation of muscle fiber endplates [98]. A secondary decline occurs between postnatal day (P)5 and P15 [99], which coincides with the time frame of neuromuscular junction (NMJ) maturation and stabilization.

Transgenic and conditional transgenic mouse models of SMA have greatly elucidated temporal and spatial requirements for SMN. For a detailed review of SMA animal models and their uses, we refer readers to Burghes et al. (2017) [100]. Briefly, increasing neuronal SMN levels (4-fold by E15) using a *prion* (*PrP*)-*SMN* cDNA transgene corrects the SMA phenotype seen in severe SMA mice [101], suggesting that elevated neuronal SMN expression is required during the perinatal developmental period. Indeed, restoring Smn expression during the early symptomatic period (P4-P8, but not P10) rescues neuromuscular pathology and motor function in mice co-expressing a ubiquitous *Cre* transgene and *Smn*-inducible alleles (*Smn^Res/Res^*) on the severe SMNΔ7 SMA background [95]. Le et al. (2011) were the first group to demonstrate the phenomenon that earlier SMN induction is most effective [102]. A tetracycline inducible expression system on the SMNΔ7 SMA mouse background was used to rescue SMN levels in SMA mice during both embryonic (E13) and neonatal periods (P0-1; P2); embryonic and early neonatal (P0-1) SMN induction robustly rescued the neuromuscular phenotype of SMA mice, while P2 induction did not [102]. Interestingly, discontinuation of SMN induction at P28 in perinatally rescued weanlings did not cause overt neuromuscular phenotypes in adulthood (one month later) [102].

These findings are supported by the work of Kariya et al. (2014), who used an inducible Cre-loxP estrogen receptor (ER) transgenic system to deplete Smn levels ubiquitously at various postnatal time points in mice homozygously expressing the *Smn^F7^* floxed allele and *SMN2* transgene; the switch from Smn requirement to insensitivity occurs abruptly at P17 [85]. This time point coincides with the end of presynaptic NMJ maturation [103] and a relative decline in the activity of Smn in assembling snRNP particles in mouse spinal cord tissue [94]. By P20, low levels of SMN (which are satisfied by two copies of *SMN2* in a mouse model) adequately maintain mature neuromuscular synapses [85]. Indeed, Smn depletion had minimal consequences throughout adulthood, except for mild neuromuscular histopathology later in life, and reduced regenerative capability in response to nerve and muscle injury [85]. Thus, increased SMN dosage is required for neuromuscular maturation, as well as regeneration or repair pathways later in life. Taken together, these studies illuminate the crucial requirement for high SMN expression during the time frame of neuromuscular maturation. The need for SMN to maintain neuromuscular integrity suggests that curative therapies should be delivered during the critical stages of neuromuscular maturation in order to prevent the establishment of irreversible defects that result in lifelong neuromuscular impairment.

Our understanding of SMN temporal requirements mostly comes from mouse studies, but perhaps the most important preclinical study comes from the porcine model of SMA, as it closely resembles SMA patients. It is the only available large animal model to study SMA pathologies and therapeutics, with a size relative to human infants [104]. The pig SMA model was created by developing a shRNA that selectively knocked down porcine SMN levels to those observed in SMA spinal cord samples. The pigs developed a clear SMA phenotype at one month of age that mirrored human SMA patients and included muscle weakness with reduced electrophysiological markers of compound muscle action potential (CMAP) and motor unit number estimation (MUNE) [104]. Rescue studies to address temporal requirements of SMN were achieved by delivering scAAV9-*SMN* the day after injection of scAAV9-shRNA targeting pig *SMN*. The pigs in this presymptomatic paradigm were completely rescued; the weakness and electrophysiological parameters were corrected. When scAAV9-*SMN* was given at symptom onset, substantial but not complete correction was observed, and there was variation in the degree of correction when the pig had more advanced symptoms. It was found that CMAP was preserved but MUNE and motoneuron counts were not. Collectively, this study demonstrated several important points: (1) that the observed porcine SMA phenotypes were SMN dependent and could be rescued if given scAAV9-*SMN* presymptomatically, and (2) major phenotypic improvements and halted disease progression may still be achieved if given scAAV9-*SMN* early in symptomology. As expected, diminished MUNE and motoneuron loss were not rescued by postsymptomatic scAAV9-*SMN* treatment in these instances, since once motoneurons have been lost they cannot be replaced. Early symptomatic treatment suggests the potential for stabilization and some NMJ sprouting to occur, as observed in this study by maintenance of the CMAP [104].

## 6. Lower α-Motoneuron Pathologies

Development of SMA mouse models has permitted critical research on SMA disease mechanisms. In particular, mouse models have allowed for the comparison of muscles that are either vulnerable or resistant to denervation, which has provided insight into the mechanisms regulating motoneuron degeneration [86,105,106,107,108,109,110,111]. SMN deficiency within the lower motoneuron circuitry induces abnormalities in SMA model mice that ultimately result in the degeneration of vulnerable spinal motoneurons and subsequent skeletal muscle weakness [112].

### 6.1. Motoneuron Somas

Early in the disease process, many cellular functions and molecular signaling pathways have been shown to be dysregulated in SMN-deficient mouse spinal cord tissue and laser-captured motoneurons. Transcripts associated with translation, rRNA binding, ubiquitin homeostasis and oxidative phosphorylation are downregulated in laser-captured, preferentially vulnerable motoneurons from the *Smn^2B/−^* mouse model of intermediate SMA at a time point prior to denervation and soma loss [113]. In addition, transcripts associated with cell death are upregulated, particularly those involved in p53 signaling [113]. Compared to laser-captured motoneurons innervating relatively resistant muscles, preferentially vulnerable *Smn^2B/−^* motoneurons downregulate transcripts associated with DNA repair [113]. Similarly, intron retention, DNA damage, and p53 pathway activation are detected in spinal cord tissue 30 days postinduction of SMN depletion in antisense oligonucleotide-based inducible SMA mice [114]. Apoptosis is not observed at this time point, indicating that these changes also precede cell death [114].

A recent study by Nichterwitz et al. (2020) [115] determined that p53 pathway activation occurs in both resistant (ocular) and vulnerable (spinal) somatic motoneurons microdissected by laser capture from SMNΔ7 mice, indicating cellular stress in both populations. However, resistant (ocular) motoneurons exhibited decreased expression of pro-apoptotic genes, increased expression of survival factors, and upregulation of pathways involved in neurotransmission, neurite outgrowth, and axon regeneration. In contrast, vulnerable (spinal) motoneurons upregulated genes related to axon degeneration and axonal transport deficits [115]. Doktor et al. (2017) performed RNA sequencing on symptomatic Taiwanese SMA mice (a severe SMA mouse model) at P5, an age preceding spinal motoneuron loss [116,117]. In this study, U12-dependent intron retention was detected in all SMA tissues examined, indicative of global splicing aberrations. Gene ontology enrichment analysis revealed downregulation of angiogenesis in all tissues (spinal cord, brain, liver, and skeletal muscle), likely reflecting growth factor depletion. Additionally, differentially expressed genes in spinal cord tissue were associated with cell division and axon guidance functions [116]. Splicing defects were assessed in greater detail by Huo et al. (2014), who used microarrays to monitor splicing at exon–exon junctions and identified strong positive and negative splicing events occurring in laser-captured lumbar motoneurons from severe SMA mice (*Smn^−/−^*; *SMN2^+/+^*) at time points just preceding spinal motoneuron death (P3-P4) [118]. These findings were validated in neuroblastoma NB2a cells subjected to RNA interference-mediated *Smn* knockdown. Differentially spliced transcripts were associated with cellular and developmental neuronal functions, transcription, and growth control, as well as RNA metabolism [118]. Interestingly, widespread alternative splicing aberrations only arise in the late stages of the disease [119]. Taken together, transcriptional profiling of SMA mouse motoneuron and spinal cord tissues highlight early alterations in a variety of molecular pathways, including those associated with neuronal development, RNA metabolism, DNA damage, and cell death.

Concurrent with these molecular signaling changes, early functional pathologies of spinal motoneurons include hyperexcitability (hyperpolarized voltage threshold for action potential firing) [86,120,121] and reduced proprioceptive synaptic input onto spinal motoneurons [86,87,120,122]. Proprioceptive afferents sense the position of muscles in relation to the trunk [123,124] and contribute significant afferent input to modulate motoneuron firing to regulate muscle tone, which is critical for postural control [125,126]. These alterations precede spinal motoneuron loss. The molecular mechanisms evoking distinct spinal motoneuron death in SMA remain unresolved [127]. While the c-Jun N-terminal kinase (JNK)/c-jun signaling axis and the p53 signaling pathway have been implicated in severe SMA mouse models [128,129,130], the molecular mechanisms carrying out spinal motoneuron loss in SMA are likely complex, potentially context dependent, and may differ based on disease severity of the SMA model [131,132].

### 6.2. Motoneuron Terminals

Insight into neuromuscular pathology was largely made possible by the advent of SMA mouse models, which have demonstrated that early neuromuscular junction (NMJ) pathology is a hallmark feature of SMA. Defects at the NMJ include neurofilament accumulation in motor axons (predominantly in its phosphorylated form) [133,134,135], and fewer active zones [136,137], synaptic vesicles [77,135,136,137], and vesicle release sensors [77,138]. In addition, motor nerve terminals have fewer mitochondria [135,137,139], which is thought to be due to defects in transport [140,141] and consequently affect microtubule maturation [137]. Other key pathologies include motor end plate immaturity [26,134,135,142], neuromuscular transmission deficits [122,135,138,143,144], dysregulated calcium homeostasis [143,145], and ultimately denervation of vulnerable motoneurons [98].

Several thorough investigations of early embryonic development using SMA model mice suggest that the neuromuscular system, even in the most vulnerable muscles, undergoes relatively normal establishment [98,146,147], indicating that the deterioration of NMJs after birth occurs in the absence of major developmental disturbances [146,147]. This evidence suggests that denervation is a consequence of a failure to maintain the synapse rather than from defective axonal pathfinding and/or endplate innervation, and that defects arise during postcontact neuromuscular maturation [134]. The extent of denervation is variable but primarily distinguishable by muscle group. For example, severe denervation (>50%) appears predominantly in vulnerable axial and appendicular muscles, while other muscles are mildly or entirely resistant to denervation at end stage disease in SMNΔ7 severe SMA model mice [98]. It is important to note, however, that patterns of denervation differ amongst severe SMA mouse models; for example, Taiwanese SMA mice develop less extensive denervation than SMNΔ7 mice [98,148]. Studies investigating motoneuron/NMJ vulnerability in SMA mouse models reported no correlation between susceptibility to denervation and muscle location, muscle fiber type, nerve bundle length, NMJ size, axonal branching patterns, pruning rates, or Schwann cell expression [98,149].

## 7. Other Cell and Tissue Types Vulnerable to SMN Deficiency

### 7.1. Skeletal Muscle

Although primary deficits in SMA are caused by motoneuron-autonomous pathologies, there is mounting evidence that intrinsic muscle defects may also occur, though this phenomenon is debated. An early study by Braun et al. (1995) demonstrated that Type I and II (but not Type III) SMA patient-derived myofibers degenerate after one to three weeks in a co-culture with wild type fetal rat spinal cord explants [150], suggesting that SMN might have a muscle-specific role that is disrupted when SMN levels are exceptionally low in these cells. Supporting this hypothesis are the observations that, firstly, the Smn complex (without any associated snRNPs) localizes to the sarcomeric z-disc in striated mouse myofibrils purified from mechanically isolated myofibers [151]. However, it is important to recognize that differences in tissue preparation methods implemented prior to immunostaining for Smn in myofibrils may impact staining (e.g., using mechanically isolated myofibers then myofibril purification vs. intact muscle cryosections). Secondly, Berciano et al. (2020) demonstrated that hypertrophied (non-denervated) human Type I SMA myofibers exhibit myofibrillar ultrastructural damage and mislocalization of SMN from I-bands and M-bands to z-discs [152]. Lastly, Kim et al. (2020) have recently shown that on the background of low human SMN expression to avoid the complete absence of Smn (which is fatal), skeletal muscle-specific Smn depletion in mice (achieved using a *MyoD-iCre* driver to diminish SMN levels similar to that in *Smn^−/−^*; *SMN2^+/+^* mice) induces morphological alterations to myofibers and NMJs, alters ex vivo force, impairs motor function by 6–7 months of age, and reduces lifespan [153]. However, an earlier study by Iyer et al. (2015) [154] used a *myogenic factor 5* (*Myf5*)*-Cre* driver to lower SMN expression in skeletal muscle to levels similar to SMNΔ7 SMA mice, thus generating higher muscle SMN levels than those used in the Kim et al. (2020) study [153] described above. They found no muscle phenotype, weakness or reduced ex vivo force production at eight weeks of age [154]. It is unknown whether neuromuscular pathologies would have developed with increased age, as no later time points were examined. Hence, skeletal muscle cells, just like all cell other types, have an inherent SMN requirement for normal function. This issue of primary muscle dysfunction will ultimately be illuminated in the future from SMA patients identified through newborn screening whom receive nusinersen treatment that is restricted to the CNS prior to symptomology versus those whom receive SMN-inducing therapies that target the whole body (described in Section 8).

Enhanced SMN expression precedes both myoblast fusion into myotubes and motor end plate innervation, raising the question of whether SMN-deficient skeletal muscle exhibits abnormal myogenesis [155]. Several in vitro studies have demonstrated impaired myogenesis induced by SMN deficiency. When Smn levels are decreased in a mouse myoblast cell line, observed defects include reduced myoblast cell proliferation and impaired myoblast fusion into multi-nucleated myotubes [156]. Fusion deficits recapitulate observations made in myoblast cultures derived from severe SMA mouse models and from Type I SMA patients (but not from individuals with milder forms of SMA) [157,158,159].

Important experimental details should be considered when interpreting studies on intrinsic muscle defects in SMA. These include species-specific differences between humans and mice, which likely include discrepancies in typical/required SMN dosage, as well as the specific SMA mouse models utilized (as each express different SMN dosages most likely due to position effects of transgene integration sites and/or promoters used). This leads to differences in SMA disease severity of functionally *Smn*-null mice homozygously expressing the transgenic *SMN2* allele. An example of this discrepancy is the transgenic *SMN2* line 89 (*TgSMN2-Ahmb89*), which is driven by the human *SMN2* promoter sequence and results in a very severe phenotype [111], versus a transgenic *SMN2* allele, which is under the control of murine *Smn* promoter and results in a mild phenotype [160].

### 7.2. Schwann Cells

A loss of non-myelinating Schwann cells may also influence neuromuscular pathology. Perisynaptic Schwann cells are reduced in number in SMA model mice in vulnerable and resistant muscles [136], fail to completely cover endplate sites [161], and express fewer key proteins required to generate the peripheral extracellular matrix [162,163]. Furthermore, selective restoration of SMN in Schwann cells improves neuromuscular function [163].

### 7.3. Astrocytes

In addition to muscles and motoneurons, SMN deficiency affects astrocytic function, which likely influences SMA pathogenesis in severe cases. Postmortem analysis of SMA patient spinal cord tissue reveals astrogliosis [164], and cultured astrocytes differentiated from SMA patient-derived iPSCs exhibit morphological and functional alterations consistent with astrocytic activation [165]. Reactive astrocytes were also observed in SMNΔ7 mouse spinal cords at ages preceding spinal motoneuron loss [165]. Critically, astrocyte-specific SMN repletion attenuates denervation, partially mitigates stripping of proprioceptive synapses onto spinal motoneurons and greatly enhances survival of *Smn^2B/−^* mice; repletion of SMN in SMNΔ7 mice also enhances survival, although this benefit was moderate [164]. Additionally, abnormal calcium regulation and reduced growth factor production has been observed in SMN-deficient astrocytes [165]. Overall, an emerging body of work indicates that low SMN levels in astrocytes located in the spinal cord may contribute to SMA disease onset and/or progression.

### 7.4. Heart

Low SMN levels may induce dysfunction in cell types beyond the neuromuscular system. In exceptional cases, which comprise infants with the most severe form of SMA (Type 0), defects in fetal cardiac development have been reported [25]. The most common abnormality is septal and cardiac outflow tract defects, which may contribute to rare but reported distal necrosis [166,167]. Additionally, benign cardiac arrhythmias have been reported in patients with milder forms of SMA [168], although these rhythmic abnormalities may be a consequence of physical inactivity and trunk muscle weakness [169]. Although anatomical and functional cardiac defects are not often observed in SMA patients, they are pervasive in severe SMA mouse models [106,170,171,172,173,174,175,176,177,178,179].

### 7.5. Additional Susceptible Cell and Tissue Types

A variable degree of thalamic dysfunction [180] and thalamic neuronal degeneration and gliosis has also been reported in severe SMA patients at end stage of the disorder [26]. Additionally, other abnormalities have been reported, including abnormalities in autonomic, sensory, gastrointestinal, and endocrine systems [22,24,26,181,182,183,184]. In animal models, a number of organ phenotypes have been noted, including abnormalities in cardiac, lymphatic, kidney, liver, pancreas, spleen, vasculature, bone and connective tissues (thoroughly reviewed in Yeo and Darras (2020) [185]). These non-canonical pathologies are generally only reported in patients with the most severe forms of SMA, suggesting that even low expression levels of SMN (achieved by 2–3 functional copies of *SMN2*) is sufficient for vitality in these tissues.

Use of SMN-based genetic therapies in humans may uncover pathology in non-motor systems, particularly in severe SMA patients who would otherwise experience a robust motor phenotype and gravely shortened lifespan that could obfuscate other organ impairments not readily apparent during the natural disease progression. Now that disease-modifying therapies are available to treat primary SMA pathologies (e.g., spinal motoneuron dysfunction and loss), secondary defects arising from chronic SMN deficiency in untargeted peripheral tissues may emerge. Future clinical research that follows Type I patients receiving FDA-approved therapies that target the central nervous system (via antisense oligonucleotides) versus the whole body (by gene therapy or oral small molecule) will shed light on the importance of SMN-deficient peripheral cell types in human SMA.

## 8. The Quest for Effective SMA Therapies: FDA-Approved SMN-Dependent Therapeutics

**Figure 4:**
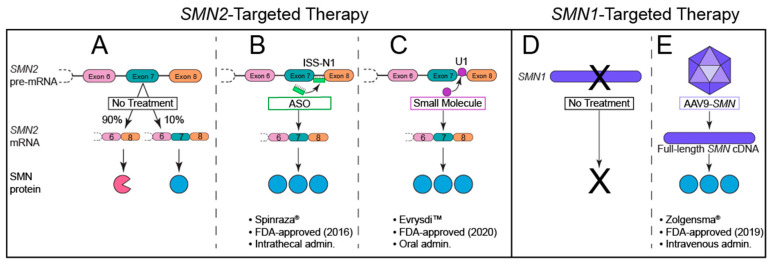
FDA-approved SMN-based therapies for SMA. (**A**) In the absence of therapeutic intervention, the *SMN2* gene produces predominantly truncated SMN protein (pink incomplete circle) and only a small percentage of full-length SMN protein (blue full circle) due to poor inclusion of exon 7 in mature mRNA transcripts. (**B**) The first FDA-approved therapy for SMA utilizes antisense oligonucleotides (ASOs; green icon; brand name Spinraza^®^) to target intronic splicing silencer N1 (ISS-N1), thereby increasing inclusion of exon 7 in mature mRNA transcripts. (**C**) The most recent FDA-approved therapy, risdiplam (purple circle; brand name Evrysdi™), enhances U1-pre-mRNA association to promote inclusion of exon 7 in mature mRNA transcripts. (**D**) SMA is caused by homozygous loss of functional *SMN1* (by gene deletion or conversion), resulting in a lack of SMN protein derived from this gene. (**E**) Onasemnogene-xioi (brand name Zolgensma^®^) causes transduced cells to transcribe full-length *SMN* cDNA.

The last decade of preclinical research searching for SMA treatments has resulted in significant advancements in our understanding of the biologic, cellular, and genetic mechanisms underlying SMA. Despite this knowledge, several challenges have made drug development difficult. SMA comprises a broad spectrum of phenotypes, with a large population developing an onset of symptoms during infancy. Additionally, therapeutic treatment must be able to effectively target disease-relevant tissue (such as lower α-motoneuron somata in the central nervous system and NMJs in the peripheral nervous system). Genotype/phenotype studies in humans and preclinical investigations have shown that the best therapeutic approach to preventing or improving disease progression is through increasing functional SMN levels. Animal model studies suggest that even a relatively modest increase in SMN, when given early enough, produces clinically meaningful improvements [104,186,187,188,189]. Notably, despite early intervention being crucial for optimal improvement, animal models suggest that restoration of SMN later in life may still provide some therapeutic benefit [95,104]. Notwithstanding these temporal and cell type-specific challenges, the first FDA-approved, SMN-based treatment became available in December 2016, and in the four years since this approval, several other promising candidates and two more FDA-approved therapies have followed (Figure 4).

Several SMN-based therapeutic approaches have been investigated to upregulate functional SMN protein. Examples of therapeutic approaches include targeting *SMN2* splicing (antisense oligonucleotides and other small molecules), transcription (histone deacetylase inhibitors, hydroxyurea, lncRNA-targeting oligonucleotides, prolactin, and quinazoline), translation (Indoprofen, aminoglycosides), as well as stabilization of SMN transcript or protein (Celecoxib) and the insertion of *SMN* genes (adeno-associated viral and lentiviral vectors) [190,191]. Currently, three SMA-modifying therapies have been approved by the FDA (detailed in the following sections), and thus far, all SMN-based approaches have similarly altered the disorder progression and outcome of SMA-affected individuals. These types of therapies are currently the best method to prevent motoneuron degeneration (if administered early enough); however, they may not be entirely curative for all patients receiving treatment. While most patients who receive presymptomatic SMN-based treatment remarkably achieve motor skills in the normal developmental range [192], there remains a population of SMA-affected individuals who would benefit from additional treatments to address persistent dysfunction. This population includes patients who have one *SMN2* copy, patients with two *SMN2* copies but experience suboptimal motor development, or patients who receive postsymptomatic treatment (when substantial motoneuron death has already occurred). These individuals will likely require additional treatment strategies to improve residual motor dysfunction.

### 8.1. Nusinersen: An Antisense Oligonucleotide (ASO)

The endogenous presence of a paralog gene that produces the necessary protein has made *SMN2* an ideal therapeutic target. If splicing of *SMN2* is corrected to produce a greater percentage of full-length SMN protein (similar to levels produced by *SMN1*), symptoms of SMA can be alleviated. The first FDA-approved therapy for SMA uses an ASO under the generic name nusinersen (brand name Spinraza^®^). Nusinersen uses a synthetic strand of nucleic acids linked together with a 2′O-methoxyethyl backbone that functions by recognizing and binding to cellular RNA to correct gene splicing. Nusinersen uses Watson–Crick pairing to specifically bind the intronic splicing silencer-N1 (ISS-N1) sequence in *SMN2*, which is a major inhibitory element regulating the splicing of exon 7. ISS-N1 has proven to be a model target for ASOs to increase the ratio of full-length SMN protein derived from *SMN2* transcripts, as ISS-N1 inhibition results in the inclusion of exon 7 in mature *SMN2* transcripts [193,194].

ASOs are endocytosed by cells upon intrathecal administration, though the mechanisms regulating absorption are poorly understood. Upon entrance to the cell body, nusinersen ASOs enter the nucleus where they bind to *SMN2* pre-mRNA transcripts to correct exon 7 splicing and thus increase full-length SMN levels (Figure 4B). Nusinersen has drastically altered the outcome and progression of this previously unmodifiable neuromuscular disorder [195], and current efforts are underway to improve ASO penetration, durability, and therapeutic safety and efficacy profiles.

Preclinical studies in mice have shown that administration of ASOs targeting ISS-N1 is sufficient to mitigate neuromuscular pathology and greatly improve survival and motor function in a time- and dose-dependent manner [173,188,189,196,197,198]. In non-human primates, a single intrathecal bolus injection of nusinersen was observed to be widely distributed throughout the spinal cord, and predominantly accumulated in small and large cell bodies of the grey matter (consistent with neural and glial cell targeting) [199]. When administered to Taiwanese Type III SMA (*Smn*^−/−^; *SMN2*^+/+^) mice, ASO-induced *SMN2* splicing changes were still detectable after six months [199], suggesting that the long half-life of the drug permits several months between treatments for SMA patients [200]. Indeed, patient autopsy results demonstrate that intrathecal delivery of nusinersen elevates *SMN2* mRNA exon 7 inclusion in neurons and other cell types in the spinal cord [201].

Clinical studies of nusinersen in all types of SMA patients showed encouraging efficacy, tolerability, and pharmacology consistent with its intended mechanism of action. Intrathecal injections were well tolerated and improved motor function in a majority of treated patients [44,46,200]. Type I SMA patients had remarkably improved lifespans compared to what would have been normally expected from the natural history of the disorder, and patients experienced fewer respiratory complications requiring ventilation [46]. More historical data are necessary to determine lifespan benefits of individuals with less severe forms of SMA. Type I SMA patients receiving nusinersen therapy achieved motor milestones that are unprecedented in the natural disorder progression, and many achieved these skills within normal time frames of motor development [46].

Patients with later-onset SMA that have received nusinersen therapy report a better quality of life, and caregivers of these patients also report a decreased impact of caring burden in addition to a greater quality of life [202]. Adult patients receiving nusinersen therapy experienced a stabilization of motor function or a reduction in symptom severity, though this improvement was not observed in all patients [203,204]. In further support of the limited benefit of delayed nusinersen treatment, Arnold et al. (2016) demonstrated that neuromuscular deficits persist in SMNΔ7 mice treated with ASOs at P4-6 [205]. The persistence of motor impairment suggests that neuromuscular function is a promising target for additive pharmacological interventions, particularly for patients that receive SMN-based treatment during adulthood.

The above treatment trials outline the effect of nusinersen treatment postsymptomatically. An interim report of the NURTURE study, an ongoing, open-label, multi-site single-arm, Phase 2 trial, which enrolled and treated SMA patients in infancy while they were presymptomatic, illustrates the importance of proactive treatment with nusinersen as soon as possible after establishing an SMA genetic diagnosis [192]. Infants enrolled in NURTURE were ≤6 weeks of age at first dose and, based on *SMN2* copy number and expected concordance of phenotype with an affected sibling(s), were predicted to have type I or II SMA (15 children with 2 *SMN2* copies and 10 children with 3 *SMN2* copies). At a median 2.9 yrs of follow up, all patients were alive without requirement of permanent ventilation, which directly opposes the natural history of untreated Type I SMA infants. Motor function was assessed using several assays, including crawling, sitting, standing, and walking (with or without assistance). All 3-copy *SMN2* patients (10/10) achieved motor milestones in timelines consistent with normal development, and all patients achieved independent sitting. A majority of 2-copy *SMN2* patients achieved motor skills unexpected in the natural history of the disease, though more difficult tasks, such as standing or walking alone, were not achieved by all children in this interim report (3/15, or 20%). However, the remaining 80% of 2-copy *SMN2* patients were able to stand or walk, though only 27–40% of patients (4/12 stand alone, and 6/15 walk alone) achieved this during normal developmental time frames. [192]. Essentially, all of the study trial result outcomes: (1) exceeded those of participants’ SMA-affected siblings who had not received this early treatment; (2) exceeded expectations based on the natural history of SMA; and (3) treatment benefits exceeded those observed when treatment is initiated in the symptomatic period. Additionally, on a molecular level, phosphorylated neurofilament heavy chain (p-NF-H) levels have been shown to be a potential biomarker of disease severity and treatment response in SMA patients who received nusinersen treatment symptomatically [192,206,207]. pNF-H is a neuron-specific cytoskeletal structural protein that is released into the CSF and plasma when axonal damage occurs [208]. The presymptomatic NURTURE trial demonstrated increased pNF-H levels at baseline patient identification [192], indicating that the disease is biologically active, warranting early treatment. Like the other trials [206,207], pNF-H levels rapidly declined postnusinersen treatment and then stabilized to a lower plateau level [192]. Collectively, these results highlight the need for early population-based newborn screening for SMA and initiation of treatment as soon as possible thereafter with SMN-inducing therapies. Furthermore, these results suggest that some 2-copy *SMN2* patients (particularly those who struggle to achieve the ability to stand or walk unassisted) would benefit from an additional, SMN-independent therapy that specifically targets neuromuscular function.

Despite immense benefits that alter the natural history of SMA, the use of nusinersen for the treatment of SMA is not without challenges. Intrathecal administration is relatively invasive, requiring sedation or anesthesia, and can be particularly problematic in young patients or patients with scoliosis. Since ASOs have a limited penetrability of the blood–brain barrier, direct administration into the cerebrospinal fluid is required to achieve endocytosis into motoneuron somata. Additionally, intrathecally administered ASOs have poor peripheral penetrance [209]; the clinical implications of which (if any) remain to be determined as nusinersen-treated individuals continue to age. Potential risks of treatment with nusinersen are thrombocytopenia and coagulation abnormalities, as well as renal toxicity [210].

In the United States, nusinersen is currently approved for the treatment of any patient with biallelic mutations or deletions of *SMN1* [211]. During the first year of treatment, nusinersen is estimated to cost $750,000 for the six recommended doses. Thereafter, the medication costs $375,000 for the recommended three maintenance doses per year. No cessation of annual treatments is recommended per se, unless the patients (or their caregivers) elect not to continue treatment.

### 8.2. Onasemnogene Abeparvovec-Xioi: A Self-Complimentary Adeno-Associated Virus (scAAV9)

The second approved therapy for SMA utilizes recombinant adeno-associated viral vectors to target the central underlying deficiency that causes SMA (Figure 4E). Onasemnogene abeparvovec-xioi (previously known as AVXS-101, and now as the brand name Zolgensma^®^, and hereafter referred to as onasemnogene-xioi) is available to patients under 2 years of age (and not exceeding 13.5 kg) with biallelic mutations or deletions of *SMN1*. Onasemnogene-xioi is a self-complementary adeno-associated viral vector serotype 9 (AAV-9) carrying human full-length *SMN* cDNA under control of the hybrid CMV enhancer/chicken β-actin promoter. Upon intravenous delivery, the non-replicating scAAV9 crosses the blood–brain barrier into the central nervous system, where it is endocytosed by cells, including motoneurons, and trafficked to the nucleus. Once in the nucleus, the virus uncoats and transduces the host cell to transcribe its double-stranded DNA unit (full-length *SMN* cDNA) [212]. This medication requires a single intravenous administration over a period of 60 min to achieve therapeutic benefit.

Targeting of cells within the central nervous system is achieved by the use of the AAV9 serotype, which has been shown to efficiently transduce neurons and glia in rodents, pigs, and non-human primates [104,187,213,214,215]. However, transduction efficiency is age dependent and varies between species and cell types. Studies in neonatal mice have demonstrated that peripheral administration of GFP-tagged scAAV9 with a chicken β-actin hybrid promoter transduced 60% of motoneurons, and was measurable 20 days after intravenous injection [215]. Using this vector for *SMN* gene replacement in SMNΔ7 neonates rescues motor function, neuromuscular physiology, and survival, while intravenous administration to P10 SMNΔ7 mice yields limited benefits [187], supporting the established crucial need for early intervention. In addition to disease progression prior to treatment, this result is also attributed to an age-related switch in transduction efficiency from neurons to glia in mice [215]. This age-dependent switch in transduction efficacy does not appear to occur in larger mammals [104,216]. Using a novel porcine model of SMA, Duque et al. (2015) elegantly demonstrated that intrathecal delivery of scAAV9-*SMN* corrected SMA phenotypes when given early (presymptomatically), but was not completely curative when delivered at symptom onset (but it did halt disease progression), due to motoneuron loss that had already occurred prior to treatment [104]. When scAAV9-*GFP* was tested in newborn (P0–P90) and 3-year-old cynomolgus macaques, systemically administered scAAV9 crossed the blood–brain barrier and robustly transduced spinal motoneurons, brain cells (in particular, glial cells), and skeletal muscle (though more so in the young animals) [213]. 3-year-old macaques had less frequent GFP^+^ expression in spinal motoneurons compared to newborns [213], although possibly due to a dosing difference. Collectively, these studies suggest that through an intravenous route, onasemnogene-xioi administration to SMA patients may have restricted benefits to phenotype when administered postsymptomatically, primarily due to inability to recover motoneurons that have already been lost.

Clinical trials of onasemnogene-xioi have demonstrated a remarkable improvement of motor function in SMA patients when treated presymptomatically. Patients with three copies of *SMN2* achieved motor skills within normal age ranges of acquisition. In comparison, a majority (but not all) of patients with two copies of *SMN2* also achieved motor milestones within normal ranges. A long-term follow up of patients who were treated postsymptomatically showed that no patients lost any achieved motor skills, which is a stark contrast to the natural history of this disorder. The most commonly achieved motor milestone included head control, rolling from back to side, and sitting unsupported for more than 30 s. However, less than half of the patients receiving postsymptomatic intervention achieved advanced motor skills such as walking or standing (supported or unsupported) [217]. Additionally, onasemnogene-xioi dramatically improved respiratory function regardless of pre or postsymptomatic administration. In comparison, over 90% of patients with severe forms of SMA not receiving SMN-based therapy require permanent ventilation by their second birthday. 

While gene therapy has remarkably changed the field of SMA treatment, use of onasemnogene-xioi is not without potential drawbacks. The inherited prevalence of maternally-derived (though potentially transient) neutralizing antibodies to AAV9 may restrict the use of this type of therapy in a minority of SMA-affected individuals due to the risk of rendering the viral vector ineffective [218,219]. Furthermore, gene transfer does not permit dose cessation if a safety issue arises. In particular, onasemnogene-xioi has a potential risk of serious liver complications, and requires monitoring of liver function prior to and for at least 3 months after infusion [220]. Thrombocytopenia and elevated troponin-I are also potential risks, requiring platelet counts and troponin-I levels to be monitored before and after treatment [220]. Additionally, unlike antisense oligonucleotides that target the endogenous *SMN2* gene(s), AAV9 vectors do not have an inherent ceiling of SMN upregulation. A final consideration should also be given to the potentially prohibitive cost of onasemnogene-xioi—at $2.1 million for a single dose, this medication is currently (January 2021) the world’s most expensive drug [221].

### 8.3. Risdiplam: An Oral, Brain-Penetrant Small Molecule

The most recently approved therapy for SMA is Evrysdi^TM^ (risdiplam), which is a daily, orally bioavailable small molecule that is brain penetrant. Similar to nusinersen, risdiplam is an *SMN2* exon 7 splice modifier (Figure 4C). This molecule promotes exon 7 inclusion by binding to two sites in *SMN2* pre-mRNA: the 5′ splice site (5′ss) of intron 7 and the exonic splicing enhancer 2 (ESE2) of exon 7. This binding stabilizes a ribonucleoprotein (RNP) complex that is critical to the specificity of this small molecule for *SMN2* over other genes [222]. Risdiplam has been approved by the FDA for all SMA patients 2 months or older, and clinical trial results show significant improvement in motor function and SMN levels, with the most notable benefits in younger patients [223,224,225]. Approximately 90% of Type I SMA patients were alive after 12 months of treatment (and reached 15 months of age or older), and none required permanent ventilation at 28 months of age [226]. Remarkably, 41% of treated Type I SMA infants achieved unsupported sitting for 5+ s [226]. Type II–III SMA patients also experienced significant and sustained improvements in motor function compared to placebo-treated controls after 12 months of risdiplam treatment [226].

Risdiplam has a list price of $340,000 per year for patients weighing 20+ kg, with the price adjusted for patients under this weight limit. Risdiplam is an attractive competitor to nusinersen and onasemnogene-xioi not just due to its lower price but also because of the convenience of at-home administration, as risdiplam can be swallowed as a flavored liquid or given in a feeding tube. Since a large percentage of SMA patients (particularly Type II SMA) experience severe scoliosis (which can complicate or deter intrathecal administration), patients, families, and health care providers may favor the use of an orally administered drug. Additionally, this method of administration permits risdiplam to be bioavailable in peripheral systems, in comparison to the limited ability of centrally administered treatments to reach peripheral organs [227]. Bioavailability in the periphery may mitigate emergence of potential secondary defects arising from SMN deficiency in peripheral tissues. More clinical research is necessary to compare the short- and long-term tolerability, safety, effectiveness, and peripheral distribution of risdiplam, nusinersen and onasemnogene-xioi therapies.

### 8.4. Cost–Benefit Considerations for Approved SMN-Based Therapies

While many American insurance companies have decided to cover SMN-based therapies, some companies have implemented stricter guidelines for patient eligibility than recommended by the FDA, such as age limitations or the requirement of symptom manifestation prior to treatment coverage [228]. These company-imposed restrictions are driven by the high drug price tag. Additionally, outside the United States, widespread approval for use of nusinersen as a therapy for SMA has been slow, primarily hampered by the high drug price [229].

For example, a recent survey indicated that insurance approval is a critical barrier to accessing nusinersen, especially among adult patients [230], as ~25% of respondents declare insurance ineligibility as one the reasons for not currently receiving nusinersen treatment. This self-reported barrier supports the notion that insurance-dictated eligibility can limit or delay treatment. In particular, adults with SMA may have already experienced significant motoneuron death, and additional delays in treatment may severely restrict any therapeutic benefit of SMN-based therapy.

The high cost of these drugs does not include the additional and substantial medical costs associated with treatment (including but not restricted to medical appointments and follow ups, administration costs, income loss due to illness or time off work, and other “invisible” costs incurred by caregivers). One survey reports that, on average, patients and/or caregivers drive 3.52 h (2 h median) to nusinersen administration centers [230], which demonstrates that significant resources (such as time and/or vehicle access requirements) are required to pursue treatment. These “invisible” costs are important to consider for health care providers and SMA-affected individuals (including caregivers) when deciding which treatment(s) to pursue.

Comparing treatment costs across patient populations is not straightforward, as two factors can influence the overall cost of medication. First, differences in dosing (single versus multiple) will dictate medical cost, though this cost may be difficult to determine as there is no recommended cessation of either risdiplam or nusinersen. Second, overall cost is also dependent on when the individual starts receiving treatment. Despite these complications in determining cost–benefit outcomes, some analyses have been made assessing the value of nusinersen and onasemnogene-xioi. A report released by the Institute for Clinical and Economic Review comparing the quality-adjusted life-year (which compares drug price tag to benefits in disease burden, assessing both quality and quantity of life) suggests that the nusinersen list price should be discounted 10-fold, and the onasemnogene-xioi list price should be discounted 2-fold [231,232]. At the time of these studies, risdiplam had yet to be approved by the FDA and was thus not evaluated. Now, with risdiplam’s FDA approval and its lower pricing, this drug may competitively drive drug costs lower for nusinersen, onasemnogene-xioi, and future disease-modifying therapies.

## 9. The Quest for Additional SMA Therapies: SMN-Independent Therapeutics

SMN-based therapies remain at the forefront for SMA treatment strategies, but not all patients receiving SMN-dependent medicine experience full symptomatic relief. Furthermore, not all individuals with biallelic *SMN1* mutations/deletions are eligible for insurance coverage of genetically targeted therapies. The risk of potentially serious adverse side effects has resulted in a debate about treating presymptomatic infants with four or more copies of *SMN2* [233,234]. However, individuals with four copies may still develop motor dysfunction [235] and thus would benefit from SMN-based therapies. Nonetheless, complex treatment decisions require parents to balance potential risks, cost, and accessibility. Additionally, insurance companies may restrict eligibility for costly SMN-based treatments, which may impact the decision of whether to treat presymptomatic individuals with 4 *SMN2* copies. Another SMA population that has been questioned for eligibility of SMN-based treatment(s) is adults that have already experienced significant motoneuron loss, who may not extensively benefit from SMN-based therapeutic approaches. For these reasons, an extensive number of SMN-independent therapeutic strategies have been investigated, which target a wide range of affected cells to protect and improve function. Ideally, these therapeutic strategies will be available to patients across the SMA spectrum and used in tandem with SMN-dependent approaches.

### 9.1. Neuroprotective Strategies

Neuroprotective strategies encompass SMN-independent treatments that are targeted to prevent dysfunction in motoneurons and associated circuitry. While several neuroprotective treatments have been previously attempted in other neurodegenerative diseases, such as Alzheimer’s disease, Parkinson’s disease, and amyotrophic lateral sclerosis (ALS), relatively poor outcomes of clinical trials have limited widespread investigation of neuroprotective agents for SMA. Neurodegeneration is a complex, multi-cellular process and, consequently, targeting single cell pathways contributing to death or survival may not be sufficient to halt or improve disease progression. Therefore, any employed neuroprotective strategies would likely require complementary SMN-targeted treatments to derive benefits.

There are two neuroprotective agents that have been approved for use in other diseases that have been tested for efficacy in SMA patients. Gabapentin, an anticonvulsant used to treat neuropathic pain and restless leg syndrome, has been evaluated due to its ability to decrease glutamate signaling and thus decrease excitotoxicity. However, two large clinical trials composed of Type II–III SMA patients demonstrated minimal effects on motor function [49,236,237]. Another drug, riluzole, has been used to treat ALS patients with some benefits. ALS patients receiving this drug have lifespans extended by 2–3 months, though with no benefit to motoneuron function. Use of riluzole in SMA animal models appeared promising, where it was able to improve neuromuscular defects in a *C. elegans* model [238], and lifespan and cytoskeletal organization in a mouse model [239]. However, a small clinical trial comprising 10 Type I SMA infants (7 riluzole-treated, 3 placebo-treated) was cut short due to lack of improvement in motor milestones, though the small cohort underpowered statistical assessment. A second clinical trial (Clinicaltrials.gov: NCT00774423) evaluated the effects of 50 mg of riluzole in Type II–III SMA patients, which has been shown to be a sufficient dose for pharmacokinetic exposure in younger SMA patients [240]. However, results of this trial have yet to be published (as of December 2020).

One neuroprotective approach that has been evaluated in SMA animal models utilizes enhancers or mimetics of neurotrophic factors. More than a dozen endogenous trophic factors have been discovered to modulate motoneuron survival in vitro, including but not limited to brain-derived neurotrophic factor (BDNF), neurotrophin-3, vascular endothelial growth factor, glial cell-derived growth factor (GDNF), ciliary neurotrophic factor (CDNF), agrin, and insulin-like growth factor-1 (IGF-1). Historically, BDNF, GDNF, and CDNF have been extensively investigated to prevent motoneuron degeneration and improve motor function [241,242,243,244,245,246,247]. However, much of the work in the SMA field has focused on IGF-1, BDNF, and agrin.

Low levels of IGF-1 have been detected in severe SMA mouse models, which are restored to normal levels upon correction of SMN levels [173,248]. Overexpression or mimetic administration of IGF-1 to SMNΔ7 mice improves lumbar motoneuron degeneration, cardiac defects, skeletal myofiber size, and motor function, but has limited benefit to survival [179,248,249,250]. Administration of AAV-driven IGF-1 into deep cerebellar nuclei of intermediate SMA model mice improves motoneuron degeneration but has no effect on neuromuscular pathology [251]. These studies indicate that IGF-1 may be a potential target for neuroprotective approaches, but is likely to have restricted benefits if used without complementary treatment(s).

The effects of BDNF have also been explored in developing motoneuron cultures and SMA models. Through its mechanism of action on TrkB receptors, BDNF application augments calcium transients via increased Cav2.2 clustering, and improves F-actin assembly and growth cone formation in motoneurons in vitro [252], suggesting that some of the developmental defects seen in SMA model cultured motoneurons [145] and mice [253] are consequences of reduced BDNF-mediated trophic support. SMN-regulated BDNF expression has also been explored in SMA model NSC-34 motoneuron-like cells. Smn deficiency results in the downregulation of the Akt signaling pathway [254], which is regulated downstream by BDNF binding to TrkB [255]. Application of loganin (a neuroprotective iridoid glycoside) to NSC-34 cells increased neurite length, cell viability, and Smn expression, upregulated BDNF and activated the Akt pathway in these cells. Loganin administration to SMNΔ7 mice improved motor function and mildly improved lifespan, and blockade of the IGF-1 receptor attenuated the protective effects of loganin [254].

The neural form of agrin (z^+^ agrin) is another trophic factor that is significantly reduced in SMA model mice [256]. Administration of NT-1654, a cleavage-resistant splice variant of z^+^ agrin with synaptogenic properties, significantly improved motor function and neuromuscular pathology [257]. The mechanism(s) regulating the reduction in trophic factors in SMN-deficient systems requires additional investigation, but nevertheless may be therapeutic targets to improve the integrity of motor circuits.

Finally, the neuroprotective agent olesoxime, which has been shown to promote neuronal survival in cultured cells deprived of trophic factors [258], demonstrated no significant benefit to Type II–III SMA patients in the OLEOS clinical trials [259]. This further supports the conclusion that neuroprotective strategies are unlikely to ameliorate motor dysfunction when used alone, and thus require a combinational approach to maximize therapeutic benefit.

### 9.2. Muscle-Directed Strategies

Given the intrinsic role of SMN in neuromuscular development, strategies that enhance muscle function have been extensively explored. One of the most investigated targets with promising results in milder SMA mouse models or as a complementary treatment to severe SMA mouse models receiving SMN-based therapy has been the inhibition of myostatin. Myostatin is a member of the transforming growth factor Β superfamily, and is predominantly synthesized and expressed in skeletal muscle. Myostatin acts as an endogenous negative regulator of skeletal muscle growth and size [260,261,262], and is neutralized by the autocrine glycoprotein follistatin and myostatin propeptide [263]. Studies evaluating the benefits of recombinant follistatin administration to SMNΔ7 mice observed improvements in lifespan, motor function, and motoneuron death [264]. However, studies directly inactivating myostatin in SMNΔ7 mice did not significantly ameliorate motor function, fiber size, or survival [265,266], likely due to the rapid disease progression in this mouse model. Nonetheless, myostatin inhibition may still provide therapeutic benefit in milder forms of the disorder and when provided in combination with SMN-based genetic therapies: one study evaluating AAV-driven myostatin inhibition in SMNΔ7 mice treated with SMN-based ASOs observed improvements in weight gain, motor function and endurance, survival, proprioceptive synapses onto motoneurons, as well as mitigated neuromuscular pathology [267]. Another study evaluating myostatin inhibition in mild SMA model mice (generated by treatment with a suboptimal dose of an *SMN2*-splicing modifier) demonstrated efficacy in late disease stages [268]. Myostatin is the therapeutic target for SRK-015, which is a monoclonal antibody that blocks the activation of the latent form of myostatin rather than inhibiting the mature myostatin form or blocking its receptor [269]. Preclinical studies in a pharmacologically induced SMA mouse model have shown that both early and late administration of muSRK-015P (a suboptimal variant of SRK-015) increases muscle mass and motor function [270]. A clinical trial is underway assessing the benefits of intravenously administered SRK-015 for Type II–III patients (Clinicaltrials.gov, NCT03921528), primarily for use as a complement to SMN-based therapy. This treatment is one of the first muscle-directed therapies to improve muscle atrophy in SMA mouse models of varying disease severity.

Another muscle-centric approach utilizes fast skeletal muscle troponin activators. Troponin complexes regulate contraction in skeletal and cardiac muscles. Fast skeletal troponin activators increase calcium affinity of the troponin-tropomyosin regulatory complex, which results in the sensitization of the sarcomere to calcium concentrations and improves skeletal muscle contractility and muscle performance. Force–calcium and force–frequency relationships are shifted leftward by troponin activation. Thus, increased contractility is observed even with inadequate neural signaling because these activators amplify the response of the muscle to submaximal nerve stimulation [271]. Reldesemtiv (CK-2127107), a second-generation fast skeletal troponin activator, has been shown to increase force production by the tibialis anterior upon transcutaneous deep fibular nerve stimulation in healthy adults [272]. Combinatorial treatment of Taiwanese SMA model mice with SMN-based genetic therapeutics and reldesemtiv enhanced the force produced by in vivo plantar flexion more than either SMN upregulator alone [273,274]; however, these preclinical data are currently unpublished. Clinical trials for *Reldesemtiv* have completed phase 2 of clinical trials (Clinicaltrials.gov, NCT02644668), but a literature summary of results have not yet been published (as of December 2020).

### 9.3. Drugs Targeting Neuromuscular Function

Despite our superficial understanding of the mechanisms regulating NMJ breakdown, a large and reproducible body of evidence supports the notion that neuromuscular junctions undoubtedly contribute to the disease pathogenesis in SMA. Reduced neuromuscular transmission and increased NMJ failure is characteristic of the SMA phenotype and has led to the testing of drugs that improve neuromuscular communication. However, few drugs have provided symptomatic relief for SMA patients. Pyridostigmine, an acetylcholinesterase inhibiter canonically prescribed to patients with myasthenia gravis, was reported to increase stamina in 2 of 4 Type II–III SMA patients [275]. Though limited, this positive finding supported the evaluation of pyridostigmine in a clinical trial comprised of patients with Type II–IV SMA living in the Netherlands (Clinicaltrials.gov: NCT02941328). While the trial was completed in 2018, results have yet to be published (as of January 2021).

Similarly, the voltage-gated potassium channel antagonist 3,4-diaminopyridine (3,4-DAP) is also undergoing two clinical trials for patients with Type III SMA (Clinicaltrials.gov: NCT03781479, NCT03819660). 3,4-DAP is canonically prescribed to patients with Lambert-Eaton myasthenic syndrome (LEMS), which is a neuromuscular disorder similarly characterized by a reduction in presynaptic calcium influx and reduced neurotransmitter release. However, dose-dependent side effects of 3,4-DAP (caused by blood–brain barrier penetration) limit symptomatic relief in patients with LEMS, so it is possible that patients with SMA will similarly experience limited benefits with 3,4-DAP. Another similar voltage-gated potassium channel-blocking drug being tested in clinical trials is 4-aminopyridine (4-AP; Clinicaltrials.gov: NCT01645787). 4-AP has traditionally been used to treat patients with multiple sclerosis due to its more efficacious penetrance of the blood–brain barrier compared to 3,4-DAP; however, similar to 3,4-DAP, 4-AP has dose-dependent side effects that restrict the use of an optimal dosage for motoneuron function. Results from these clinical trials have also not yet been published [49]. Similar to other SMN-independent approaches discussed in this review, these neuromuscular-targeted medications are unlikely to immensely benefit SMA patients when used as a stand-alone therapy. In particular, these drug interventions occur after the critical period of neurotransmission-regulated NMJ development, and thus are unlikely to reverse established neuromuscular defects [276]. However, if neuromuscular-targeted medicines are utilized during the critical period of NMJ development, they may provide long-term and substantial therapeutic benefits, as described below.

One recent investigation by Tejero et al. (2020) [253] investigated the effect of (*R*)-Roscovitine, a cdk-5 inhibitor with positive allosteric effects on voltage-gated calcium channels (Cav2.1-Cav2.2), on SMA pathology during motoneuron development. Application of (*R*)-Roscovitine to SMA motoneuron cultures increased Cav2.2 channel clustering, spontaneous calcium transients, elongated axons, and improved neurotransmission [253]. Axonal elongation was similar when motoneuron cultures were exposed to a derivative of (*R*)-Roscovitine, GV-58, which is a molecule with more potent Cav2.1-2.2 effects and no significant cdk activity at physiological ATP levels [277]. Additionally, systemic administration of (*R*)-Roscovitine to pregnant dams (E11.5-17.5) significantly increased the lifespan of SMNΔ7 mice. This lifespan benefit was independent of effects on cdks, as (*S*)-Roscovitine (which lacks Cav2 activity but retains cdk-5 inhibition) did not improve lifespan [253].

Another preclinical study evaluated the effect of GV-58 alone, or in combination with 3,4-DAP, which has been shown to synergistically increase neuromuscular transmission in Lambert Eaton myasthenic syndrome-affected NMJs [278]. The use of both GV-58 with 3,4-DAP restored neuromuscular transmission to control levels in ex vivo neuromuscular junctions in SMNΔ7 mice, but GV-58 alone was sufficient if the mice had been treated with ISS-N1 ASOs at birth. Similar results were observed in vivo, when measuring changes in muscle strength after an acute subcutaneous administration of GV-58 and 3,4-DAP to P10 SMNΔ7 pups. Untreated SMNΔ7 mice maximally benefited from the combination of GV-58 and 3,4-DAP, while ASO-treated SMNΔ7 mice maximally benefitted from GV-58 alone (with no additive benefit of 3,4-DAP) [279,280]. Results from these studies suggest that targeting calcium homeostasis in developing motor nerve terminals may remarkably alter neuromuscular dysfunction and enhance motor ability, and can be used to complement SMN-dependent approaches.

### 9.4. Endogenous SMN-Independent Protective Modifiers

An attractive target for additive therapies includes endogenous disease modifiers, but the last decade of research has produced controversial and perplexing observations. Family studies indicate that the *SMN2* gene is the main modifier of the SMA phenotype, where more *SMN2* copies equate to a milder phenotype, but this observation is not absolute, and other modifiers exist both within and outside of the *SMN2* region [9,11,12,281,282,283,284,285,286,287,288,289,290,291,292,293,294,295,296]. Transcriptome-wide differential expression analysis of genes from SMA-discordant families have elucidated endogenous protective modifiers of the SMA phenotype in some individuals, but these modifiers are not ubiquitously associated with phenotypic improvement across all SMA patients [282,294,297] Curiously, several of these putative modifiers function by sensing or regulating calcium. These positive modifiers include plastin 3 (PLS3) [80,290,298], neurocalcin delta (NCALD) [299], and calcineurin-like EF-hand protein 1 (CHP1) [300].

Some have proposed that the downregulation of the neurocalcin delta (NCALD) gene is a protective modifier of the SMA phenotype. NCALD functions as a neuronal calcium sensor to negatively regulate endocytosis. Heterozygous knockdown of NCALD improves endocytosis in fibroblasts derived from SMA patients. Knockdown also improves axon elongation and NMJ size in severe and mild SMA model mice, and accelerates neuromuscular maturation and improves motor function in intermediate SMA model mice. Additionally, suppression of NCALD improves proprioceptive contacts onto motoneuron somas [299]. A dual approach to treating SMA has been evaluated by combining SMN-increasing ASOs and NCALD-reducing ASOs in severe SMA model mice. Compound muscle action potentials, motor unit numbers, muscle fiber size, and grip strength were preserved when these two treatments were combined, compared to the use of SMN-ASOs alone [301]. Whether the endocytosis alterations (and thus ASO uptake into cells) resultant from NCALD downregulation contributes to the phenotypic improvement is unclear, however. In contrast to these positive modifying results, one investigation observed no association of NCALD and phenotypic modification unless multiple mutations of NCALD coincided to possibly create a cryptic splice site [294]. Thus, it is possible that NCALD-regulated modification is not widely applicable to most SMA-affected individuals with discordant phenotypes to *SMN2* copy numbers.

PLS3 was the first reported positive modifier of the SMA phenotype, and was found to be highly upregulated in differentiated motoneurons obtained from fibroblasts of discordant siblings [290]. This report found that high levels of PLS3 protected individuals with 3–4 copies of *SMN2* from SMA onset even in the presence of biallelic *SMN1* deletion [290]. However, other studies debate the applicability of PLS3 protection across a large patient population. One recent publication utilized next-generation sequencing to evaluate PLS3 variants and found no correlation of PLS3 variants and phenotype, suggesting that PLS3-driven phenotypic modification may only occur in a small population of patients [297].

PLS3 is located on chromosome Xq23 and is a calcium-dependent F-actin-bundling protein that modulates the cytoskeleton, axonal growth and migration, vesicle trafficking, endocytosis, and regulates the ratio of G-actin to F-actin [302,303,304,305]. One study observed that overexpression of PLS3 improved the survival of mild and severe SMA model mice [298,302,306], while overexpression in SMN-deficient motoneurons and SMA morpholino zebrafish restored axonal growth and motor function [290,302,307]. It is noteworthy to repeat, however, that axon growth defects are not observed in SMA model mice [146,147] and thus putative modifiers likely do not alter axonal growth in humans. At the level of the spinal cord, PLS3 overexpression increased motoneuron soma size and the number of proprioceptive synapses in SMA model mice [80]. At the NMJ, PLS3 upregulation corresponded with augmented neurotransmission [80], restored endplate and muscle fiber size, improved vesicle trafficking and nerve terminal accumulation, restored endocytosis and actin dynamics, and increased the number of terminal active zones of SMA model mice and zebrafish [80,298,307]. Additionally, PLS3 was found to stabilize synaptic innervation, resulting in delayed axonal pruning in NMJs of SMA mice [80], thus improving the weakened nerve–muscle connection characteristic of SMA. Additionally, a study performed by Alrafiah et al. (2018) also observed reduced axonal defects in PLS3-upregulated motoneuron cultures, as well as improvements in lifespan of SMNΔ7 mice after PLS3 upregulation, although they did not observe sustained improvement in weight gain [302]. In contrast to these variable but positive benefits, however, an investigation by McGovern et al. (2015) observed no benefit to neuromuscular function, weight, lifespan or phenotype after PLS3 upregulation in SMNΔ7 mice [308]; Bowerman et al. (2009) also did not observe phenotypic improvement associated with elevated PLS3 levels [81]. Interestingly, Kaifer et al. (2017) observed benefits of PLS3 upregulation in mild SMA mouse models but not in severe SMNΔ7 mice [306]. The debate regarding PLS3 as a protective modifier remains unresolved, and further studies are required to understand which mechanism(s) or pathway(s) associated with PLS3 may modify the SMA phenotype.

PLS3 has several binding partners, one of which is the calcineurin inhibitor CHP1. CHP1 dephosphorylates proteins involved with calcineurin phosphatase activity and has elevated expression in SMA model mice. Knockdown of CHP1 restored axonal growth in Smn-depleted NCS34 motoneuron-like cells, SMA model zebrafish, and primary SMA model mouse motoneuron cultures [300]. In SMA model mice treated with SMN-based ASOs, CHP1 reduction prolonged survival, improved electrophysiological defects, NMJ growth and maturation, and muscle fiber size in comparison to the effects of ASOs alone. In addition to CHP1, PLS3 also binds to coronin-1C (a protein encoded by the *CORO1C* gene) in a calcium-dependent manner to mediate endocytosis and actin dynamics [298]. This evidence suggests that actin dynamics in motoneurons is a calcium-dependent process that strongly modulates disease pathogenesis by increasing neuromuscular function and stabilizing motoneuron circuitry.

### 9.5. Physical Therapy Strategies

The benefits of exercise have been evaluated in SMA model mice and SMA-affected individuals. In intermediate SMA model mice, elevated levels of full-length *Smn* have been observed after either acute or chronic exercise [309,310]. Chronic exercise significantly improved motoneuron maturation and soma loss in the lumbar spinal cord [311] and extended lifespan [310], suggesting that exercise can mitigate pathology and disease progression. Elevated levels of IGF-1 have been observed after exercise in SMA mice, potentially providing neuroprotective support through a trophic action rather than through muscle size improvement [312]. However, these trophic and SMN upregulation benefits were not observed in Type II patients performing arm cycling exercises [313]. Other studies evaluating the benefits of physical exercise in mild SMA model mice found improvements in glucose homeostasis, oxygen consumption, and muscle mitochondria function [314], especially when the exercise was high intensity. Chronic exercise in these mice improved muscle fatigue, neuromuscular excitability, and increased the resistance of muscles to damage [315].

Evaluation of exercise in patients has been primarily reported on Type II–III SMA patients not receiving SMN-based therapies. Rehabilitative interventions for SMA-affected individuals include physical therapy, strengthening and balance exercises, aquatic therapy, and physical activity. Most published reports on the benefits of exercise have been individual case studies [316], although some clinical trials have been initiated. For example, cycle ergometer training in Type III SMA patients has been demonstrated to improve oxidative capacity but induces significant fatigue [317]. Other programs have utilized at-home strength and aerobic exercise trainings to improve motor function, strength, fatigue, and cardiovascular fitness in patients. Benefits of these exercises (in particular, aerobic exercise) have been difficult to assess due to a high drop-out rate [318]. However, exercise through sport activity has been shown to significantly improve self-esteem and identity, reduce depression, and result in a greater quality of life for patients with neuromuscular disorders, including SMA-affected individuals [319]. With new FDA-approved SMN-based therapies improving the ability of SMA-affected individuals to participate in activity that demands endurance, strength, and motor skills, exercise may be an excellent, low-cost and accessible method to improve the negative psychological and emotional aspects of SMA.

### 9.6. Biomedical Devices

Advances in biomedical devices have shown that some SMA-affected individuals may benefit from use of orthoses (externally applied devices designed to improve muscular function), such as robotic exoskeletons. These devices improve quality of life for people with muscular diseases [320,321,322,323,324], including SMA. One study demonstrated positive effects on range of motion and performance of daily living activities of SMA-affected individuals [322]. Orthotic-assisted enhancement of motor function correlated with higher self-esteem, increased participation in school, and more social interaction [322], which result in a better quality of life. Another biomedical device, the robotic stretcher, leverages the retained function of limited digit movements to improve the operation of a motorized wheelchair, thus bettering independent maneuvering [325]. These devices have the ability to improve motor function for individuals who either do not experience significant improvement of motor function through other therapeutic means or are unable to utilize other treatments due to established weakness or paralysis, or due to lack of access. It will be interesting to see how these devices might further benefit the SMA population when used in conjunction with SMN-based genetic therapies.

## 10. Future Directions for SMA Therapies

The long-term success of SMA therapy depends on the extent of improvement and permanency of recovery. For most individuals, the best therapy to salvage motor function and lifespan will be SMN dependent. This conclusion is supported by compelling and vast evidence from animal models and patients demonstrating that SMN is crucial for motoneurons, and that restoration of SMN at any time point can provide (albeit sometimes limited) benefits to motor function. Current FDA-approved therapies for SMA (nusinersen, onasemnogene-xioi, and risdiplam) do not fully rescue motor impairment or development for all patients, particularly those who have only one copy of *SMN2* or receive postsymptomatic treatment. Additionally, patients who are either ineligible for, or do not receive, SMN-based treatment (due to cost, availability, access, or condition) will require SMN-independent strategies to improve quality of life through better motor function.

Muscle strength and endurance (and consequently motor skill) are driven by neuromuscular activity. Motor skills permit the performance of activities required for daily living, including wheelchair mobility, daily tasks such as food preparation or hygienic practices, and the use of a keyboard and mouse. The ability to perform these activities would provide meaningful clinical improvement to patients and their caregivers [35,38]. SMN-based therapy is a remarkable start to improving motor function, but patients that respond suboptimally to treatment would benefit from complementary, SMN-independent medicine to further improve motor skills.

### Finding a Cure through Complementary Treatment: NMJs Are Crucial Targets

Numerous reports demonstrate that NMJ instability is a crucial component of SMA pathogenesis [85,122,135,143,145,147,253,257,326,327], and evidence suggests that neuromuscular weakness persists after SMN-based treatment [46,279,280,327,328,329,330]. The potential discovery of SMA phenotypic modifiers further support the notion that the protection of NMJs can significantly modify disease progression [290,299,300]. Additionally, embryonic intervention to protect developing NMJs can improve the postnatal phenotype [253], suggesting that perinatal development is a critical window for optimal therapeutic benefit.

There remains a gap in effective SMA therapies that directly protect neuromuscular function despite the potential to drastically improve patient fatigue, independence, and quality of life [38]. For patients who receive suboptimal motor benefits from SMN-based medicines, protecting and improving neuromuscular function will be critical. One of the populations most vulnerable to persistent NMJ dysfunction after SMN-dependent treatments are adult SMA patients, as it is likely that some motoneuron loss has already occurred by the time SMN-based treatment is initiated.

One recent study that supports the need for NMJ-targeted treatment evaluated neuromuscular function after nusinersen treatment of adult SMA patients. Arnold and colleagues utilized repetitive nerve stimulation (3 Hz) and measured compound muscle action potential (CMAP) decrement to determine neuromuscular function. The investigators found that a large proportion of ambulatory and non-ambulatory adult SMA patients had improved CMAP amplitudes after 10–14 months of nusinersen treatment, but maintained a CMAP decrement (>10% at 3 Hz) upon repetitive nerve stimulation [330], indicating that neuromuscular transmission defects persist after nusinersen treatment. Additionally, the investigators observed a correlation between CMAP decrement and motor function (measured using 6 min walk test and fatigue, elbow extension/flexion, shoulder abduction and revised upper limb module). These data suggest that neuromuscular defects in adult SMA patients constitute a secondary pathology that is not improved by SMN-based strategies. One possible explanation for this observation is that neuromuscular denervation persists after SMN restoration but can be compensated for by collateral sprouting. Additionally, the investigators did not observe a correlation between CMAP decrement and disease severity (ambulatory or non-ambulatory function), duration of disease (years since symptom onset), or patient age [330]. Thus, individual differences in NMJ transmission may be an SMN-independent modifier of disease phenotype [330]. Understanding the mechanisms underlying neuromuscular dysfunction will be crucial for restoring motor ability and strength in patients receiving SMN-based therapy.

Similar results investigating neuromuscular pathology before and after ISS-N1 ASO treatment have also been observed in SMNΔ7 mice. One study noted a similar persistence of CMAP decrement (measured during adulthood) after P4 ASO treatment in SMNΔ7 mice [330]. Another study demonstrated that P0-1 ASO treatment of SMNΔ7 mice did not restore neuromuscular transmission in a highly vulnerable muscle (transverse abdominis). Furthermore, the investigators also observed that increased presynaptic transmitter release (via drug-induced augmented calcium influx into motor nerve terminals) in vivo resulted in greater in vivo muscle strength in SMNΔ7 mice [279,280], further supporting the notion that neuromuscular transmission correlates with motor function in SMA. Together, these studies indicate that established neuromuscular pathology is unlikely to be reversed by SMN-based medicine and thus complementary therapies are needed to optimally stabilize and strengthen neuromuscular connections and thus maximize motor ability. This goal might be achieved by use of drugs that increase presynaptic neurotransmitter release, alter postsynaptic excitability, and/or enhance response to neurotransmitter, which can increase neurotrophic support [331] as well as increase the number of NMJs firing for a particular stimulus rate and consequently induce stronger muscle contractions.

As individuals receiving SMN-based therapy(s) progress past natural disorder outcomes, new insight will potentially reveal previously obfuscated or residual pathologies that require targeted treatments to improve quality of life. In order to develop a therapeutic cure for SMA, complementary SMN-dependent and -independent treatment strategies are necessary to address all aspects of SMA pathology to improve quality of life across the lifespan of SMA-affected individuals.

## Figures and Tables

**Table 1:** Natural histories of Type 0–Type IV SMA.

SMA Type	Age of Symptom Onset	Defining Motor Function	Common Characteristics	Life Expectancy	*SMN2* Copy Number
Type 0	Prenatal	Respiratory support	- Reduced movement in utero - Respiratory intervention usually needed from birth - Severe muscle weakness and hypotonia - Areflexia - Facial diplegia - Joint contractures - May have widespread systemic dysfunction	˂6 months	1
Type I	0–6 months	Never sits unsupported	- Poor head control - Unable to sit without support - Paradoxical breathing; respiratory failure - Muscle weakness and hypotonia - Areflexia or hyporeflexia - May have bulbar involvement	˂2 years	2
Type II	6–18 months	Sits; never stands independently	- Progressive muscle weakness and hypotonia - Hyporeflexia - Respiratory dysfunction - Musculoskeletal abnormalities - Polyminimyoclonus	>2 years	2–3
Type III	˃18 months	Walks	- Progressive muscle weakness and hypotonia - May lose ability to walk - May develop polyminimyoclonus	Adult	3–4
Type IV	˃21 years	All motor function	- Very mild but progressive muscle weakness and hypotonia - Gait abnormalities	Adult	≥4
